# Neuroimmune Interactions in Neurodegeneration: The Role of Microglia in Alzheimer’s and Parkinson’s Disease Pathogenesis

**DOI:** 10.3390/brainsci16020154

**Published:** 2026-01-29

**Authors:** Pradeep Goyal, Lalji Baldaniya, Lalit Kumar Tyagi, Kamal Kant Joshi, Suhas Ballal, A. Sabarivani, Subhashree Ray, Deepak Nathiya, Ashish Singh Chauhan, Monica Gulati, Tapan Behl, Ansab Akhtar

**Affiliations:** 1 Saraswati College of Pharmacy, Gharuan, Mohali 140413, Punjab, India; golupharma1982@gmail.com; 2 Marwadi University Research Center, Department of Pharmaceutical Sciences, Faculty of Health Sciences, Marwadi University, Rajkot 360003, Gujarat, India; lalji.baldaniya@marwadieducation.edu.in; 3 Lloyd School of Pharmacy, Plot No. 11, Knowledge Park II, Greater Noida 201306, Uttar Pradesh, India; lalit.tyagi@lloydcollege.in; 4 Department of Allied Science, Graphic Era Hill University, Dehradun 248002, Uttarakhand, India; kamal_josi@yahoo.com; 5 Centre for Promotion of Research, Graphic Era Deemed to be University, Dehradun 248002, Uttarakhand, India; 6 Department of Chemistry and Biochemistry, School of Sciences, JAIN (Deemed to be University), Bangalore 560041, Karnataka, India; b.suhas@jainuniversity.ac.in; 7 Department of Biomedical, Sathyabama Institute of Science and Technology, Chennai 600119, Tamil Nadu, India; sabarivanibme@gmail.com; 8 Sharda School of Pharmacy, Sharda University, Greater Noida 201310, Uttar Pradesh, India; 9 Department of Biochemistry, IMS and SUM Hospital, Siksha ‘O’ Anusandhan (Deemed to be University), Bhubaneswar 751003, Odisha, India; subhashreeray@soa.ac.in; 10 Department of Pharmacy Practice, NIMS Institute of Pharmacy, NIMS University Rajasthan, Jaipur 303121, Rajasthan, India; dnathiya@nimsuniversity.org; 11 Division of Research and Innovation, Uttaranchal Institute of Pharmaceutical Sciences, Uttaranchal University, Dehradun 248007, Uttarakhand, India; ashishchauhan.pharmacy@gmail.com; 12 School of Pharmaceutical Sciences, Lovely Professional University, Phagwara 1444411, Punjab, India; monicagulati14@gmail.com; 13 ARCCIM, Faculty of Health, University of Technology Sydney, Ultimo, NSW 20227, Australia; 14 Amity Institute of Pharmacy, Amity University Punjab, Mohali 140306, Punjab, India; 15 LSU Health Sciences Canter, School of Medicine, New Orleans, LA 70112, USA

**Keywords:** Parkinson’s disease, Alzheimer’s disease, microglia, neuroimmune, immune response

## Abstract

Neuroimmune interactions play a critical role in the pathogenesis of neurodegenerative disorders such as Alzheimer’s disease (AD) and Parkinson’s disease (PD), with microglia acting as key mediators of neuroinflammation. Microglia exhibit dual roles, contributing to both neuroprotection and neurotoxicity depending on their activation state. In AD, amyloid-beta (Aβ) aggregation leads to chronic microglial activation, resulting in excessive pro-inflammatory cytokine release (e.g., TNF-α, IL-1β, IL-6), oxidative stress, and synaptic dysfunction. In PD, α-synuclein aggregation triggers a similar neuroinflammatory cascade, exacerbating dopaminergic neuronal loss in the substantia nigra. Beyond inflammatory responses, microglia regulate synaptic plasticity, phagocytose pathological proteins, and interact with peripheral immune cells, influencing disease progression. Emerging evidence suggests that genetic variants in genes such as *TREM2*, *CD33*, and *HLA* modulate microglial function, thereby altering susceptibility to neurodegeneration. Dysregulated microglial responses, characterized by impaired clearance of protein aggregates and prolonged neuroinflammation, further amplify neuronal damage. Therapeutic strategies targeting microglial activation are under investigation, aiming to balance neuroinflammatory responses and enhance clearance mechanisms. Small-molecule inhibitors, monoclonal antibodies, and modulators of innate immune pathways are being explored to mitigate microglia-driven pathology. Understanding the complex interplay between microglia and neurodegeneration could pave the way for precision medicine approaches, optimizing treatments based on individual immune profiles. Further research is essential to delineate microglial heterogeneity across disease stages and uncover novel targets for therapeutic intervention.

## 1. Introduction

A class of long-term, progressive illnesses known as neurodegenerative diseases (NDs) is defined by the progressive death of neurons in both the peripheral and central nervous systems. Numerous functional impairments, including memory loss, difficulties with movement, and other neurological problems, are brought on by this degeneration. Alzheimer’s disease, Parkinson’s disease, Huntington’s disease, and amyotrophic lateral sclerosis (ALS) are noteworthy examples of NDs [1,2,3]. About 703 million individuals worldwide were afflicted by these diseases in 2019, and estimates suggest that as the population ages, this figure is projected to increase to 1.5 billion by 2050 [4]. In addition to affecting one’s physical health, neurodegenerative conditions have a significant negative influence on society and medical facilities. Major neurological conditions led to 349.22 million Disability-Adjusted Life Years (DALYs) and 10.06 million deaths worldwide in 2019 [5]. The load varies by region, with some places experiencing considerable decreases as a result of increased knowledge and access to healthcare. Instead of halting or reversing the course of the disease, current ND therapies mostly concentrate on symptom management. Among the available treatments are drugs that reduce symptoms without addressing the underlying reasons. Novel strategies, including gene therapy, stem cell therapy, and nanotherapeutics, are being researched as possible paths to better medical care. Drug distribution and effectiveness [5] are still hampered by issues like the blood–brain barrier. Neurodegenerative conditions are a major worldwide health concern that has a big impact on both the afflicted person and society as a whole. The goal of ongoing research is to understand the mechanisms underlying these ailments and create more potent treatment plans to help millions of people throughout the world.

Alzheimer’s disease is the most prevalent neurodegenerative disorder worldwide, while Parkinson’s disease represents the second most common neurodegenerative disease. Accordingly, Alzheimer’s and Parkinson’s diseases remain the two most extensively studied neurodegenerative disorders due to their prevalence, progressive nature, and substantial societal impact [6]. AD is distinguished by the progressive death of neurons that results in a marked deterioration in cognitive and motor function. Whereas PD is characterized by the loss of midbrain dopaminergic neurones, which causes tremors and decreased movement [7]. Although amyloid plaques correlate with cognitive impairment in AD, clinical trial evidence shows that their removal alone does not robustly slow cognitive decline. Anti-amyloid antibody trials demonstrate substantial reductions in brain amyloid burden, yet only modest clinical benefit, particularly when treatment is initiated after significant synaptic and neuronal damage, highlighting limitations of plaque-targeted therapies in advanced disease stages [8,9,10,11]. Current treatments for many disorders merely provide symptomatic relief without changing the course of the disease, resulting in significant human suffering and financial costs. Despite a growing emphasis on creating disease-modifying medications that target underlying mechanisms including neuroinflammation and oxidative stress, no ground-breaking treatments have yet to be developed. It is essential to comprehend the unique pathophysiological characteristics of AD and PD in order to develop treatment approaches and enhance patient outcomes [5].

The pathophysiology of NDs, which are marked by progressive neuronal loss and cognitive deterioration, is significantly influenced by inflammation and immunological responses. Studies show that chronic inflammation, which is frequently made worse by stress, age, and dysfunction of the mitochondria, causes an imbalance in immunological responses, especially when microglia are activated and pro-inflammatory cytokines like TNF-α and IL-1β are released [12]. In addition to causing neuronal damage, this neuroinflammatory process also starts a feedback loop that makes inflammation worse and further disrupts brain function [13]. Furthermore, as indicated by new gene therapy approaches targeted at modifying inflammatory responses, the interaction between neuroinflammation and dementia implies that targeting immune pathways may have therapeutic promise [14]. Therefore, creating effective treatments for these severe diseases requires a knowledge of the complex relationship that exists between inflammation and neurodegeneration.

Because they are involved in both neuroinflammation and neuroprotection, microglia are essential to the pathophysiology of both AD and PD. Amyloid-β buildup and tau hyperphosphorylation are two variables that worsen the neurodegeneration caused by hyperactive microglia in AD, which results in chronic inflammation and poor neurogenesis [15,16]. Pro-inflammatory mediators may be released as a result of dysregulated microglial activity, further harming neuronal cells. While earlier literature frequently described microglial activation in terms of pro-inflammatory (M1-like) and anti-inflammatory (M2-like) phenotypes, this binary framework is now understood as a simplified heuristic rather than a stable biological classification. Contemporary evidence instead supports microglial activation as a continuum of dynamic, context-dependent functional states that shift with disease stage, regional vulnerability, and environmental cues. The distinct functions of M1 and M2 microglia in PD are crucial; M1 microglia promote inflammation and neuronal damage, whereas M2 microglia facilitate tissue regeneration and debris removal [17]. Developing tailored therapeutics that reduce the impact of neuroinflammation in these disorders requires an understanding of these pathways.

Current research on the involvement of microglia in neurodegenerative conditions indicates that these cells can both contribute to disease pathology and exert essential homeostatic functions. Microglia are critical for maintaining brain homeostasis by regulating inflammatory signaling, clearing cellular debris through phagocytosis, and modulating synaptic remodeling. In Alzheimer’s disease, microglia additionally influence neurogenesis by regulating neural progenitor cell dynamics through neurotrophic factor secretion and activity-dependent synaptic pruning [18]. Nevertheless, microglial activation in neurodegenerative settings can result in persistent inflammation and neurotoxicity, and different subpopulations show different patterns of gene expression associated with either neuroprotection or neurodegeneration. Understanding the intricate variability of microglial responses is one of the challenges, since the pathogenic environment and peripheral immune cells can affect the activation states of these cells. There are still unanswered questions regarding the exact processes by which microglia change from protective to detrimental roles, underscoring the necessity of focused treatment approaches to successfully alter their activities. Throughout this review, it is important to distinguish findings derived from experimental models of Alzheimer’s and Parkinson’s diseases from pathological processes occurring in the human conditions. Animal models, cellular systems, and induced pluripotent stem cell-based approaches reproduce selected aspects of disease biology but do not fully recapitulate the temporal progression, cellular complexity, or heterogeneity of human neurodegeneration. Accordingly, observations described in experimental models are discussed as mechanistic insights that inform disease-related pathways rather than as direct representations of pathological changes in patients. Despite extensive investigation into neuroinflammation, a unifying framework that establishes microglia as essential pathogenic drivers across neurodegenerative diseases remains lacking.

This review is significant because it integrates recent genetic, molecular, and functional evidence to position microglial dysfunction as a central determinant of disease initiation and progression in both Alzheimer’s and Parkinson’s diseases. By critically synthesizing findings from experimental models, human genetics, biomarker studies, and therapeutic interventions, this review moves beyond descriptive cataloging to highlight shared and disease-specific microglial mechanisms that actively shape neurodegeneration. Clarifying these convergent pathways is essential for guiding the development of microglia-targeted therapeutic strategies with improved translational relevance.

## 2. Microglia: Overview and Function in a Healthy Brain

Embryonic development of the CNS begins in the third week of pregnancy, predominantly from the ectoderm, which differentiates into the neural ectoderm, generating the neural tube that eventually develops into the brain and spine. This process, known as neurulation, is crucial because the neural tube shuts by the fourth week, and following brain structures, including as the prosencephalon, mesencephalon, and rhombencephalon, emerge during the fifth and sixth weeks. The CNS continues to mature after birth, providing it susceptible to environmental effects and genetic abnormalities that can cause deformities such as spina bifida and microcephaly [19]. Recent advances in optical recording methods have improved our understanding of embryonic CNS function, revealing intricate neural network activity throughout early development [20,21]. Thus, a thorough understanding of embryonic CNS development is required for detecting and treating congenital abnormalities.

Microglia are the CNS’s resident immune cells, playing critical roles in brain homeostasis, immune response, and neurogenesis. Understanding their origins and development is critical to understanding how they work in health and illness. Microglia develop from early embryonic erythromyeloid progenitors (EMPs) located in the extraembryonic yolk sac. This happens during primitive haematopoiesis, when these progenitors move into the developing embryo and colonise the rudimentary brain. According to studies, microglial progenitors develop in yolk sac blood islands and travel to the neuroepithelium, where they divide and differentiate into microglia [19].

## 3. Physiological Functions of Microglia

Microglia are immune cells that conduct critical physiological processes necessary for brain health and neuronal integrity. They have a role in controlling neuronal excitability and synaptic plasticity, especially in areas such as the hypothalamus, where they interact dynamically with neurones to regulate electrical responses and maintain homeostasis. Furthermore, microglia use tunnelling nanotubes to allow the interchange of organelles and proteins with neurones, which is critical for rescuing neurones from toxic aggregates linked to neurodegenerative disorders. The roles they play include coordinating immune responses and tissue regeneration, emphasising their relevance in neurodevelopment and neurodegeneration [22]. Furthermore, microglia adjust their activities in response to environmental cues, revealing a complex interaction between their states and the surrounding neuronal context, which is essential for successful neuroprotection and recovery after damage [23].

### 3.1. Surveillance of the Brain Environment

Microglia play an important part in brain environment monitoring, undergoing dynamic morphological and functional alterations in response to changes in neural circumstances. Recent studies using miniature two-photon microscopy (mTPM) have shown that microglial surveillance is closely related to brain state transitions during sleep–wake cycles, with significant changes observed during sleep deprivation, such as phagocytic activity and morphological contraction [24]. The locus coeruleus–norepinephrine system has been identified as critical for mediating these microglial responses, emphasising the role of neurotransmitter signalling and microglial activity in maintaining sleep homeostasis [23,24]. Additionally, by clearing debris from the environment and promoting cellular communication, microglia help maintain tissue homeostasis, which is essential for the CNS growth and immunological response. This complex function emphasizes how crucial microglial monitoring is for the brain’s ability to adjust to both normal and pathological changes [25].

### 3.2. Synaptic Pruning and Maintaining Homeostasis

Microglia are crucial for maintaining homeostasis and synaptic pruning in the CNS. They actively aid in the enhancement of brain circuits, which is essential for synaptic plasticity and neural network maturation, by phagocytosing excess or weaker synapses [26]. Because microglia aid in the establishment of vital hypothalamic circuits that control glucose homeostasis, this pruning process is especially important during early development [27]. Furthermore, by eliminating extra neuroblasts and apoptotic cells, microglia preserve homeostasis while promoting healthy neurogenesis and averting disease [18]. Microglia can prune astrocytes in response to external stimuli, such as drug exposure, which alters glutamate homeostasis and behavior. This phagocytic activity is also linked to these reactions [28]. Therefore, microglia play a crucial role in the brain’s general homeostatic balance as well as synaptic refinement.

### 3.3. Role in Neuroprotection and Repair

In a variety of neurological disorders, such as spinal cord injury and ischemic stroke, microglia are essential for neuroprotection and healing. By controlling immunological responses, encouraging neurogenesis, and bolstering metabolic processes, they preserve brain homeostasis and aid in healing. For example, microglia help with locomotor recovery following spinal cord damage by improving lipid metabolism, which is essential for neuronal function, and creating glial scarring [18]. Microglia have two roles in ischemic stroke: they first aid in tissue regeneration by removing debris and attracting oligodendrocyte precursor cells, but their persistent activation may aggravate damage and cause neuroinflammation [29]. Furthermore, microglia mitigate oxidative stress and restore function in neurodegenerative conditions by transferring healthy organelles to damaged neurons via tunnelling nanotubes [30]. Comprehending these intricate roles of microglia is crucial for creating personalised treatments meant to improve their neuroprotective potential.

## 4. Microglia in Immune Responses

As the brain’s innate immune protectors, microglia are the main resident immune cells of the CNS. They are essential for immunological responses as well as preserving homeostasis in the central nervous system. An outline of their roles in immunological responses, activation processes, and cell-to-cell contacts is provided below.

### 4.1. Innate and Adaptive Immunity

In the CNS, microglia serve as the first line of defence against infections and damage. By changing from a quiescent to an active state, when they carry out a variety of immunological tasks, including phagocytosis and antigen presentation, they may react quickly to infections or injury. Major Histocompatibility Complex (MHC) class I and II molecules are expressed by activated microglia, which allows them to deliver antigens to T cells and connect innate and adaptive immunity. In both healthy and diseased CNS conditions, microglial activation is essential. Under normal circumstances, microglia remain dormant, carrying out housekeeping tasks and expressing few cytokines. To control inflammation, they become activated in response to stimuli like debris or misfolded proteins, taking on a phagocytic function and generating pro-inflammatory cytokines. Microglia can change into “primed” microglia, which are less able to recover to a protective state and contribute to persistent inflammation and neuronal damage, in pathological situations, especially in neurodegenerative disorders like Alzheimer’s [31]. A range of microglial activation states that can either promote or impair neuronal health result from this dysregulation, which is made worse by environmental variables and genetic predispositions [32]. Certain biomarkers linked to these activation states have been found in recent research, which might help track the course of the disease and guide treatment decisions [33].

### 4.2. Cytokine Production

A crucial part of the immune response, cytokine production is impacted by a number of variables, such as infections, immunomodulators, and radiation. Numerous cytokines secreted by activated microglia have the ability to regulate inflammation [34]. Among them are pro-inflammatory cytokines, such as TNF-α and IL-1β, which increase inflammation and draw more immune cells to areas of damage or infection. On the other hand, they also generate anti-inflammatory cytokines, which aid in tissue healing and inflammation reduction [12,34].

### 4.3. Activation Mechanisms

#### 4.3.1. Toll-like Receptors (TLRs)

Numerous TLRs (TLR1–9) that are expressed by microglia are able to identify pathogen-associated molecular patterns (PAMPs). When different agonists stimulate these receptors, microglia are activated and produce more cytokines and chemokines, which improve their capacity to respond to immunological stimuli. For example, bacterial membrane lipopolysaccharides (LPS) activate TLR4, but bacterial cell walls’ peptidoglycan activates TLR2 [35].

#### 4.3.2. Phagocytosis

Activated microglia engulf pathogens and cellular detritus as phagocytic cells. This process involves the release of growth factors that promote neuronal survival and regeneration, in addition to eliminating toxic substances. Maintaining the health of the CNS, particularly in neuroinflammatory situations, requires the capacity to phagocytose. Beyond inflammatory signaling, phagocytosis represents a fundamental mechanism through which microglia and astrocytes influence AD progression. Microglia actively internalize and degrade amyloid-β aggregates, tau species, synaptic elements, and apoptotic neurons through receptor-mediated phagocytic pathways involving TREM2, complement receptors, and scavenger receptors. Impairment of microglial phagocytic capacity results in ineffective clearance of pathological substrates and contributes to the accumulation of neurotoxic protein aggregates. In parallel, astrocytes contribute to debris and synapse clearance via phagocytic receptors such as multiple EGF-like domains 10 (MEGF10) and MER Proto-Oncogene, Tyrosine Kinase (MERTK), and dysfunction of astrocytic phagocytosis further exacerbates synaptic loss and neuronal vulnerability. Together, disrupted glial phagocytic activity links neuroinflammation to progressive neurodegeneration in Alzheimer’s disease [36,37,38].

#### 4.3.3. Communication with Other Cells

In the CNS, microglia interact intricately with neurons, astrocytes, and other cell types. They facilitate synaptic pruning and preserve the integrity of brain circuits by directly monitoring neuronal activity through their activities. Microglia interact with peripheral immune cells that have infiltrated under pathological situations in order to coordinate an extensive immune response. In conclusion, because of their many roles in phagocytosis, inflammation control, and communication with other brain cells, microglia are essential for both short-term immune responses to challenges in the CNS and long-term brain health maintenance. They can adapt to a variety of pathogenic problems thanks to their capacity to transition between distinct functioning states.

Although multiple microglial mechanisms including Toll-like receptor signaling, cytokine release, inflammasome activation, phagocytosis, and synaptic pruning, have been implicated in neurodegenerative disorders, their pathogenic relevance differs substantially across AD and PD, and not all observed changes represent primary disease drivers. Strongly supported mechanisms are those supported by convergent evidence from human genetics, longitudinal biomarkers, and causal perturbation studies. In AD, this includes microglial lipid sensing and phagocytic pathways involving TREM2, PLCG2, and APOE, which modify amyloid burden, tau propagation, and disease risk in humans. Similarly, in PD, genetic and experimental evidence supports a causal role for microglial responses to misfolded α-synuclein, particularly via inflammasome-linked and phagocytic pathways that precede dopaminergic neuron loss. In contrast, cytokine elevations and broad inflammatory signatures, while reproducibly observed in both diseases, often correlate with disease stage and neuronal injury rather than initiating pathology. These responses likely represent amplifying or downstream processes, rather than primary triggers. Likewise, activation of pattern recognition receptors such as TLRs is context-dependent and may reflect sensing of existing protein aggregates rather than disease-specific pathogenic signaling. Importantly, disease specificity must be emphasized. Complement-mediated synaptic pruning is strongly implicated in early AD-related synapse loss but has less direct evidence in PD, whereas α-synuclein-driven microglial activation and phagocytic overload are central to PD but not primary features of AD. Collectively, these distinctions highlight that not all microglial changes are equally pathogenic. Differentiating causal, disease-specific mechanisms from epiphenomenal inflammatory responses is essential for accurate interpretation of experimental findings and for the rational development of microglia-targeted therapies.

## 5. M1 vs. M2 Phenotypes and Their Respective Roles in Immune Responses

M1 and M2 microglia are historically defined activation phenotypes that have been used as a simplified framework to describe opposing pro-inflammatory and anti-inflammatory immune responses. The pro-inflammatory characteristics of M1 microglia include the release of cytotoxic substances that worsen neuronal damage, especially in disorders like spinal cord injury and PD [17]. However, accumulating transcriptomic and functional evidence indicates that this binary M1/M2 classification does not fully capture the dynamic, heterogeneous activation states of CNS-resident microglia in chronic neurodegenerative diseases. By releasing cytokines that aid in the removal of debris and suppress inflammatory reactions, M2 microglia, on the other hand, demonstrate anti-inflammatory properties that support tissue repair and neuroprotection [32]. Modulating microglial inflammatory and reparative signaling pathways, often interpreted within the M1/M2 framework, has demonstrated therapeutic promise in experimental models. For example, transplanting M2 microglia edited with miR-145a-5p has improved recovery in SCI models by preserving M2 features and inhibiting M1 activation. Importantly, such interventions likely influence specific microglial functional programs such as phagocytosis, metabolic regulation, or cytokine signaling rather than inducing stable M1 or M2 phenotypes. Furthermore, substances such as Apilarnil have shown promise in restoring M1/M2 polarization, which reduces motor dysfunction in PD models [39]. Developing effective treatments for neurodegenerative diseases therefore requires understanding microglial activation as a continuum of context-dependent functional states, rather than as a fixed M1/M2 polarization [33,39]. In order to improve neuroprotection and lower inflammation in neurodegenerative diseases, recent research has looked into therapeutic approaches to alter this polarization. These include the use of low-intensity pulsed ultrasound and different nutraceuticals, which have demonstrated promise in reorienting microglia from the M1 to the M2 phenotype [39]. Accordingly in AD and PD, the M1/M2 paradigm is best understood as a pedagogical heuristic. Microglial activation is described in modern models as a dynamic continuum of reversible states, including disease-associated, metabolic, and interferon-responsive programs shaped by regional and age-dependent heterogeneity, which is best defined through transcriptomic and functional profiling rather than fixed polarization [40]. Throughout this review, M1/M2 terminology is retained only for historical and pedagogical clarity, and all mechanistic interpretations are grounded in modern transcriptomic, functional, and disease-stage-specific models of microglial activation.

## 6. Neuroimmune Interactions in Alzheimer’s Disease

AD is the most common kind of dementia, making about 60% to 80% of cases worldwide. Its incidence significantly rises with age, especially after age 65. AD’s pathogenesis includes the buildup of neurotoxic beta-amyloid plaques and hyperphosphorylated tau neurofibrillary tangles, which results in neuronal degeneration and is characterized by memory impairments, cognitive decline, and behavioral abnormalities.

Amyloid-beta (Aβ) plaques and tau neurofibrillary tangles are the two main pathogenic characteristics that accumulate in AD. Clarifying the processes underlying AD and creating successful treatments requires an understanding of how these two elements interact. Aβ is made up of 36–43 amino acid peptides that are mostly produced when beta-secretase and gamma-secretase enzymes cleave amyloid precursor protein (APP) [41]. The Aβ40 and Aβ42 variants are especially important because Aβ42 is more hydrophobic and amyloidogenic, which contributes to its main involvement in plaque development. Amyloid plaques are harmful to neurons and are formed when Aβ peptides assemble. Although amyloid plaques are a defining pathological hallmark of Alzheimer’s disease, accumulating experimental evidence indicates that insoluble plaque deposits are not the primary neurotoxic species. Instead, soluble amyloid-β oligomers exert greater synaptotoxic and neurotoxic effects by disrupting synaptic signaling, impairing neuronal calcium homeostasis, and triggering microglial activation. Amyloid plaques may act as reservoirs that sequester soluble amyloid-β species, whereas plaque-associated microglial responses and local inflammatory signaling contribute indirectly to neuronal injury. This distinction helps explain why substantial plaque removal does not uniformly translate into robust cognitive improvement [9,10,42]. Neurodegenerative processes, like synaptic dysfunction and cell loss, are linked to these plaques [43]. Tau pathology is thought to be caused by a series of processes that are started by Aβ. It causes normal tau protein to change into a toxic form, which aids in the development of neurofibrillary tangles inside neurons. This relationship implies that Aβ serves as a “trigger” or precursor in the pathophysiology of AD [44,45]. In addition to synaptic dysfunction, progressive neuronal loss is a defining pathological feature of AD and closely correlates with disease severity and cognitive decline. Neurodegeneration in AD prominently affects hippocampal and cortical neurons and is driven by the combined effects of amyloid-β toxicity, tau pathology, chronic neuroinflammation, and oxidative stress. Importantly, microglial-mediated inflammatory signaling and impaired clearance mechanisms contribute directly to neuronal vulnerability and cell death, reinforcing the central role of neuroimmune interactions in AD progression [46,47].

**Figure 1:**
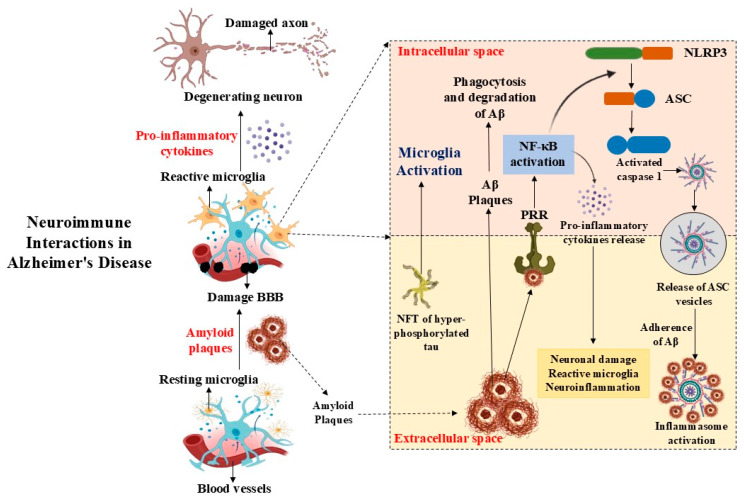
Neuroimmune interaction in Alzheimer’s Disease. BBB: Blood–Brain Barrier; NLRP3: NLR family pyrin domain containing 3; Aβ: Amyloid-beta; NF-κB: Nuclear factor-kappa B; ASC—apoptosis-associated speck-like protein.

Neuronal microtubules are stabilized by the microtubule-associated protein tau. Tau aggregates into tangles inside neurons as a result of hyperphosphorylation in AD. This mechanism leads to cell death by interfering with microtubule activity. Tau and Aβ have a reciprocal interaction; whereas Aβ causes tau toxicity, abnormal tau can intensify Aβ toxicity by means of feedback mechanisms [43]. This synergy implies that both proteins play a key role in promoting AD development. Developing treatment methods requires an understanding of the interplay between tau and Aβ. According to recent studies, both tau and Aβ diseases must be identified early in order to prevent serious cognitive impairment. The visualization of these medical conditions in living individuals is made possible by imaging techniques like PET scans, which offer insights into the course of the disease and possible locations of intervention. A key factor in the onset and progression of Alzheimer’s disease is the interaction between tau pathology and amyloid-beta pathology [41]. In order to improve outcomes for those who are at risk for or have been diagnosed with AD, ongoing research attempts to elucidate their functions in greater detail and investigate targeted therapeutics that may lessen their detrimental consequences (Figure 1).

### 6.1. The Role of Aβ Plaques and Tau Tangles in Initiating Neuroinflammation

Tau and Aβ both play important roles in the pathophysiology of AD by causing neuroinflammation, which is a major factor in the development of the condition. Aggregation of Aβ peptides, which can cause neuroinflammatory reactions, results in Aβ plaques. Microglia and astrocytes, two glial cell types involved in the brain’s immunological response, are activated when Aβ builds up in the brain. Pro-inflammatory cytokines like TNFα and IL-1β are released as a result of this activation, and they can worsen the damage to neurons and synaptic dysfunction [12]. Beyond its role as a downstream inflammatory mediator, IL-1β directly promotes amyloidogenic processes by transcriptionally upregulating the expression of APP. Experimental studies in neuronal and glial systems demonstrate that IL-1 signalling enhances APP gene expression and amyloid-β production, thereby establishing a feed-forward loop in which neuroinflammation actively drives amyloid pathology rather than merely responding to it. This mechanistic link provides strong evidence that chronic microglial-derived IL-1β signalling contributes directly to AD pathogenesis by coupling inflammatory activation to increased amyloid burden [48,49]. Aβ aggregates have been shown to interact with pattern recognition receptors on astrocytes and microglia, triggering an innate immune response that increases the release of reactive oxygen species and inflammatory mediators. Neurodegeneration is thought to be caused by this dysregulated immune response. Further tying inflammation to AD pathophysiology, systemic inflammation has been demonstrated to promote Aβ accumulation in the brain [15,16].

Another factor linked to neuroinflammation is tau tangles, which are produced when tau proteins become hyperphosphorylated. According to studies, tau oligomers can bind to microglia and astrocytes, increasing neuroinflammatory activity [12] Compared to Aβ plaques, the presence of NFTs correlates more strongly with cognitive impairment, indicating that tau disease may affect neuronal health directly. It is interesting to note that tau itself can affect the production of Aβ; in several animal models, it has been demonstrated that tau deletion of genes inhibits the generation of Aβ and the formation of plaque [12]. This interaction between tau and Aβ points to a complicated connection in which both substances affect each other’s pathogenic processes in addition to contributing to neuronal toxicity. Tau tangles and Aβ plaques are important contributors to the development and maintenance of neuroinflammation in Alzheimer’s disease. The neuronal damage and cognitive impairment seen in AD patients are mostly caused by the combination of these two disease characteristics. Developing tailored therapeutics to reduce neuroinflammation and halt the course of disease requires an understanding of these pathways.

Importantly, accumulating evidence from human studies substantiates a central role for microglia in neurodegenerative disease pathophysiology. Genome-wide association studies have identified risk variants in microglia-enriched genes, including TREM2 and PLCG2, that significantly modify susceptibility to Alzheimer’s disease. In parallel, cerebrospinal fluid biomarkers such as soluble TREM2 reflect microglial activation states and are associated with disease stage, brain atrophy, and cognitive trajectories in patients. Neuroimaging studies using translocator protein positron emission tomography further demonstrate that microglial activation emerges early in both Alzheimer’s and Parkinson’s diseases and correlates with subsequent neurodegeneration. Postmortem analyses of human brain tissue confirm sustained microglial activation in affected regions, reinforcing that microglial dysfunction observed in experimental models reflects disease-relevant processes in humans [50,51,52,53].

### 6.2. Microglial Activation in AD

Accumulating experimental, genetic, and biomarker evidence indicates that microglia are not merely reactive responders in AD but constitute an essential pathogenic component of disease initiation and progression [52]. Insights from experimental models are interpreted here in the context of converging evidence from human genetic, biomarker, and neuroimaging studies. Alterations in microglial function precede overt neuronal loss and cognitive decline, and genetic perturbations selectively affecting microglial signaling pathways are sufficient to modify disease trajectory [54]. These findings support a model in which microglial dysfunction actively drives amyloid-β accumulation, tau propagation, synaptic loss, and neurodegeneration, rather than arising solely as a secondary consequence of neuronal pathology [36]. Evidence from experimental models of Alzheimer’s disease indicates that microglia exert context-dependent pathogenic and compensatory functions through interactions with amyloid-β and tau pathology. By interacting with astrocytes, which improve Aβ clearance and maintain neuronal health, microglia can phagocytose Aβ, lowering its toxicity and fostering neuroprotection [55]. On the other hand, pro-inflammatory reactions brought on by excessive microglial activation can worsen tau pathology and synaptic loss, which in turn might contribute to cognitive decline. In particular, the clearance of neurotoxic DAMPs, including Aβ and hyperphosphorylated tau, is mediated by microglial receptors; however, their malfunction might hinder this process, increasing the seeding and spread of tau surrounding plaques [56]. Therefore, figuring out how microglial activation and control balance out is essential to understanding how they affect the development of AD.

Experimental studies demonstrate that A1 astrocytes, induced by microglia-derived inflammatory signals, acquire neurotoxic properties, whereas A2 astrocytes promote neuronal survival and tissue repair [57]. Furthermore, some lipid classes are linked to inflammatory processes, and microglia are implicated in the dysregulation of lipid metabolism in AD [58]. This suggests a complicated interaction between microglial activity and metabolic alterations in the disorder. Therefore, focusing on microglial polarization and activation offers a possible therapy option for AD. Some of the recent studies carried out to analyze role of microglia in AD are listed in Table 1.

While the studies summarized in Table 1 provide strong evidence for altered microglial activity in AD models, it is important to distinguish microglia-intrinsic mechanisms from secondary responses to broader neuroinflammation. Several interventions, such as modulation of Egln3 or APOE4-associated microglial programs, appear to act directly on microglial signaling and metabolism, whereas others may reflect indirect effects driven by systemic inflammation or neuronal pathology. Furthermore, most findings derive from transgenic or in vitro systems, and replication across independent models remains limited. Translational constraints include species-specific differences in microglial gene expression, disease stage-dependent microglial states, and the challenge of selectively targeting microglia in the human brain without affecting peripheral myeloid cells.

Pathological stimuli like lipopolysaccharides frequently cause microglia to become activated, which results in the release of extracellular vesicles carrying S-nitrosylated proteins that spread neuroinflammatory signals and the generation of nitric oxide (NO) [64]. Chronic neuroinflammation is also associated with immunoproteasome dysregulation, which can worsen neurodegeneration by presenting self-antigens and extending inflammatory states. Therefore, a possible treatment approach for reducing neurodegenerative disorders is to target microglial activation and associated inflammatory pathways [65]. Consistent with this concept, repeated low-intensity innate immune stimulation can induce a microglial tolerance program that suppresses subsequent amyloid-β oligomer-evoked inflammatory activation and cytokine release, suggesting that innate immune memory states may be therapeutically leveraged to reduce Aβ-driven neuroinflammation [66]. Neuroinflammation and oxidative stress are tightly interconnected processes that reinforce one another during neurodegeneration. Activated microglia generate reactive oxygen and nitrogen species through NADPH oxidase and mitochondrial dysfunction, leading to oxidative damage of lipids, proteins, and nucleic acids. In turn, oxidative stress amplifies inflammatory signaling by activating redox-sensitive pathways, including NF-κB and NLRP3 inflammasome signaling, thereby sustaining microglial activation. Experimental studies demonstrate that excessive microglial-derived reactive oxygen species not only exacerbate amyloid-β and tau pathology but also impair neuronal antioxidant defenses, creating a feed-forward cycle that accelerates synaptic loss and neuronal degeneration in AD [67,68,69,70,71].

In AD models, microglia-derived IL-1α, TNF-α, and C1q have been shown to drive astrocytes toward a neurotoxic reactive state, resulting in impaired synaptic support and increased neuronal vulnerability. Conversely, astrocytic signaling pathways regulate microglial recruitment, clustering around amyloid plaques, and efficiency of amyloid clearance, indicating that microglial responses are contingent on astrocytic state and function. Genetic or pharmacological disruption of this microglia-astrocyte communication axis alters plaque-associated inflammation, synaptic integrity, and neuronal survival, underscoring that glial contributions to neurodegeneration arise from coordinated, interdependent cellular programs rather than isolated microglial or astrocytic actions [57,72,73].

It is becoming more well acknowledged that microglial participation in synaptic dysfunction in AD plays a crucial role in the development of the disease. Excessive levels of microglial activation markers, such as TREM2, have been linked to synaptic dysfunction, especially when amyloid-β pathology is present [74]. It has been demonstrated that changes in microglial dynamics occur before cognitive loss in animal models, with a reduction in Iba1-positive microglia associated with compromised synaptic integrity and hippocampus network function [75]. Additionally, through mechanisms involving complement factors such as C1q, activated microglia can drive aberrant synaptic pruning and synapse loss, contributing to cognitive impairment. In human AD brain tissue, synapses containing tau oligomers have been shown to be preferentially engulfed by microglia and astrocytes, supporting a direct glial contribution to synaptic pathology [76]. All things considered, the findings emphasize the dual function of microglia in processes that impact synaptic health in AD in both positive and negative ways, underscoring their potential as therapeutic targets. It is becoming more well acknowledged that microglial participation in AD-related neurodegeneration plays a crucial role in the development of the disease. Further linking microglia to AD pathophysiology, genetic research shows that microglial clones with pathogenic mutations, specifically impacting the MAPK pathway, trigger a potent neuro-inflammatory response [77]. A mediating role in cognitive decline is also shown by studies employing translocator protein (TSPO) PET imaging, which shows that microglial activation develops before to tau pathology and neurodegeneration. The significance for targeted treatment approaches is highlighted by these findings, which emphasize the dual function of microglia in AD, where their activation can both worsen and perhaps lessen neurodegenerative processes.

Recent advances in single-cell and spatial transcriptomic technologies have fundamentally reshaped understanding of microglial heterogeneity in AD, revealing transcriptionally distinct microglial states that are not apparent from bulk analyses. Single-nucleus RNA sequencing of human AD cortex has identified disease-associated microglial populations characterized by upregulation of genes involved in lipid metabolism, phagocytosis, and innate immune signaling, with these states emerging prior to extensive neuronal loss. Importantly, these transcriptomic programs show marked sex-dependent differences: female brains exhibit stronger enrichment of inflammatory and interferon-responsive microglial signatures, whereas male brains display greater representation of microglial states associated with metabolic stress and phagocytic pathways. Experimental manipulation of these sex-biased microglial programs alters amyloid burden and synaptic vulnerability in a sex-specific manner, indicating that microglial transcriptional state and biological sex interact to shape disease trajectory. These findings highlight the necessity of incorporating transcriptomic resolution and sex as biological variables when interpreting microglial contributions to AD and when designing microglia-targeted therapeutic strategies [78,79,80].

Microglial involvement in AD-related neurodegeneration is strongly influenced by environmental stimuli. According to research, environmental variables including exposure to chemicals and systemic microbial infections can activate microglia, hence intensifying neurodegenerative processes. For example, research indicates that in transgenic mice models of AD, a “dirty” environment accelerates up the loss of cortical neurons, emphasizing the function of microglial activation in response to pathogen-associated molecular patterns (PAMPs) [81]. While transgenic and toxin-based models of AD and PD have provided indispensable mechanistic insight, important limitations must be acknowledged. In particular, transgenic strategies can introduce unintended disruptions of endogenous genes or confounding effects from transgene regulatory elements. A well-characterized example is the rTg4510 mouse model, in which tauopathy-like neurodegeneration arises not only from overexpression of mutant human MAPT but is also influenced by additional genetic factors, including deletion of the endogenous Fgf14 locus and expression of the tetracycline transactivator (tTA) transgene. These factors independently modulate neuronal excitability, neurodegeneration, and disease severity, complicating attribution of pathological outcomes solely to tau accumulation. Such findings highlight that construct validity-faithful recapitulation of a disease-associated molecular lesion does not necessarily ensure predictive validity, as therapeutic responses in these models may not reliably forecast efficacy in humans [82,83]. Accordingly, results derived from transgenic AD and PD models should be interpreted with caution and validated across complementary systems, including human genetic, biomarker, and postmortem data, to strengthen translational relevance. Furthermore, both amyloid beta and tau pathologies, which are crucial in AD, progress due to prolonged immunological stimulation and neuroinflammation driven by malfunctioning microglia. Also, exposure to toxicants such as lead acetate during development has been associated with altered microglial responses, especially in females, indicating that early environmental variables may predispose people to AD by compromising microglial function [84]. Importantly, while multiple inflammatory pathways are activated in AD, current evidence most strongly supports microglial dysfunction in lipid metabolism, phagocytosis, and synapse regulation as disease-modifying processes, whereas generalized cytokine elevation likely reflects secondary amplification of ongoing neurodegeneration.

### 6.3. Microglial Targeting in AD Therapy

Given the critical role that microglial cells play in the pathophysiology of AD, addressing them in treatment has drawn a lot of interest. Microglia are dysregulated in AD, which leads to persistently neuroinflammatory environments that worsens neuronal damage [85]. This dysregulation is typified by unchecked microglial activation, which encourages tau pathology and β-amyloid buildup, two important characteristics of AD [44]. As a result, treatment approaches that modify microglial activity are being investigated as possible ways to slow the course of AD.

Numerous opportunities for microglial-targeted treatments are highlighted by recent studies. Therapeutic antibodies targeting triggering receptor expressed on myeloid cells 2 (TREM2) act through extracellular mechanisms and do not require entry into the cytoplasm of microglia. TREM2 is a transmembrane receptor expressed on the microglial cell surface, and antibody binding modulates receptor signaling externally [86]. Preclinical and clinical pharmacokinetic studies demonstrate that systemically administered TREM2 antibodies cross the blood–brain barrier at low but pharmacologically sufficient levels, primarily via nonspecific transcytosis, allowing engagement of microglial surface TREM2 within the central nervous system. Importantly, these antibodies do not cross the plasma membrane; instead, they exert their effects by stabilizing TREM2 signaling, enhancing microglial survival, metabolic fitness, and phagocytic capacity [86,87,88]. This extracellular mode of action underscores the feasibility of TREM2 immunotherapy while highlighting the importance of receptor-level modulation rather than intracellular antibody delivery.

Importantly, prior investigations demonstrate that “microglia targeting” is highly dependent on timing, disease substrate (amyloid vs. tau), and the specific pathway manipulated. For example, sustained depletion of microglia using a brain-penetrant CSF1R inhibitor (PLX5622) in a 5×FAD model markedly altered plaque pathogenesis, including suppression of parenchymal plaque development and broad reversal of disease-associated transcriptional signatures, but also revealed potential trade-offs such as altered Aβ deposition patterns consistent with vascular redistribution [89]. In tauopathy, pharmacologic CSF1R inhibition reduced pathogenic tau and shifted non-microglial gene-expression patterns toward a healthier signature; however, functional rescue and survival benefits showed sex-dependent effects, highlighting biological heterogeneity that must be explicitly considered in translational design [80].

TREM2-directed strategies similarly illustrate that amplifying microglial pathways can be beneficial in some windows yet detrimental in others. Microglia-specific elevation of TREM2 reduced amyloid seeding and neuritic dystrophy when applied during early stages of plaque establishment, supporting a time-limited therapeutic window for enhancing microglial barrier and clearance programs [90]. In contrast, chronic antibody-driven TREM2 activation in an Aβ amyloidosis context exacerbated Aβ-associated tau seeding/spreading and neuritic plaque pathology without robust plaque reduction, emphasizing that sustained “activation” can intensify downstream tau-linked neurodegeneration depending on context [91]. Finally, early clinical translation of microglia-receptor agonism is now feasible: a first-in-human evaluation of the TREM2 agonistic antibody AL002 demonstrated target engagement and measurable pharmacodynamic biomarker changes in CSF/plasma, supporting clinical testability while underscoring the need for mechanism-specific safety monitoring and stage-appropriate patient selection [92]. Together, these prior studies argue that next-generation microglia therapies should be framed not as global suppression/activation, but as state- and stage-specific reprogramming with explicit stratification by pathology stage and biological modifiers.

Furthermore, research has shown that microglia are not classified as M1 (pro-inflammatory) or M2 (anti-inflammatory), but rather display a range of activation levels [93]. Because of this intricacy, treatment strategies should focus on regulating microglial responses rather than just inhibiting their activity. In animal models of AD, for instance, pharmacological manipulation of Kv1.3 channels has demonstrated promise in lowering neuroinflammation and amyloid deposition [94]. Accordingly, references to M1- or M2-like states in therapeutic contexts should be interpreted as descriptive shorthand for dominant inflammatory or reparative signaling programs, rather than as discrete, stable microglial phenotypes.

There remain difficulties with microglial targeting in AD treatment, despite its potential. Finding universal therapeutic targets is made more difficult by the variability of microglial populations, as various states may play diverse roles in inflammation and neurodegeneration. Minimizing off-target impacts also requires selective targeting that does not impact other CNS cell types.

#### Microglial Contributions to the Mechanism of Action of Anti-Amyloid Antibodies

Recent clinical and mechanistic evidence indicates that the therapeutic effects of anti-amyloid monoclonal antibodies are critically dependent on microglial engagement rather than passive amyloid solubilization alone. Lecanemab and Donanemab preferentially bind aggregated amyloid-β species and form immune complexes that are recognized by Fcγ receptors expressed on microglia, thereby promoting antibody-dependent cellular phagocytosis of amyloid deposits. In vivo imaging and postmortem analyses from treated individuals demonstrate increased clustering of activated microglia around amyloid plaques following antibody administration, consistent with Fc-mediated microglial recruitment and clearance activity [95,96].

Importantly, human genetic studies further support a microglia-dependent mechanism of action. Variants in microglial genes involved in phagocytosis and immune signaling, including TREM2 and FCGR2A, have been shown to modify amyloid plaque removal efficiency and downstream biomarker responses in antibody-treated patients, indicating that intact microglial function is required for optimal therapeutic efficacy [97,98]. Consistent with this, cerebrospinal fluid biomarker analyses from Donanemab-treated patients reveal reductions in downstream tau pathology that correlate more closely with markers of microglial activation than with absolute plaque removal, suggesting that microglial-mediated remodeling of the plaque microenvironment contributes to disease modification [99].

Together, these findings demonstrate that anti-amyloid antibodies act in part by reprogramming microglial responses toward enhanced phagocytic and barrier functions, rather than solely by reducing amyloid burden. This microglia-dependent mechanism provides a biological explanation for the modest but reproducible slowing of cognitive decline observed in clinical trials and underscores the importance of microglial state, genetic background, and disease stage in determining therapeutic outcomes.

## 7. Neuroimmune Interactions in Parkinson’s Disease

Aggregation of alpha-synuclein (α-Syn) is a key factor in the pathophysiology of Parkinson’s disease (PD) [100]. The condition is marked by aberrant α-Syn accumulation in the form of Lewy bodies and Lewy neurites, as well as the loss of dopaminergic neurons, especially in the substantia nigra. Rather than being passive residue, these aggregates are linked to a number of neurotoxic mechanisms, such as lysosomal dysfunction, mitochondrial impairment, and synaptic disruptions, that lead to neuronal malfunction and death [101]. Because of its remarkable conformational flexibility, α-Syn may take on a variety of structural shapes, including fibrils, protofibrils, and oligomers. Regarding neurotoxicity and neural network propagation, each of these types demonstrates unique characteristics. Some of the recent studies carried out to analyze role of microglia in PD are listed in Table 2.

Collectively, these studies highlight consistent directionality of microglial involvement in PD models; however, critical limitations remain. In several cases, including exercise and immunomodulatory approaches, the observed effects likely reflect combined actions on microglia and other immune or neural cell types rather than microglia-exclusive mechanisms. Replication across distinct genetic and toxin-based models is variable, and few studies directly assess microglial specificity using cell-targeted approaches. Moreover, translational challenges include differences between acute experimental models and the chronic, progressive nature of human PD, as well as uncertainties regarding long-term modulation of microglial activity in patients.

**Figure 2:**
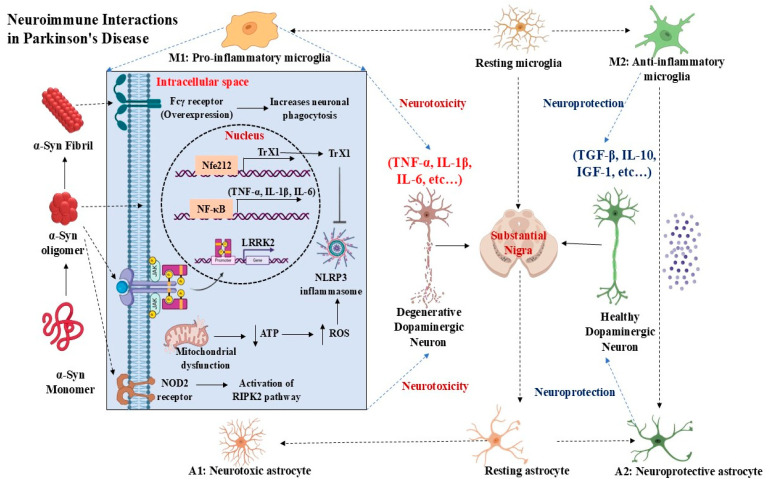
Neuroimmune Interactions in Parkinson’s Disease NF-κB: Nuclear factor-kappa B; ATP: Adenosine triphosphate; ROS: Reactive Oxygen Species; NLRP3: NLR family pyrin domain containing 3; TNF-α: tumor necrosis factor-alpha; IL-1β: interleukin-1-beta; IL-6: interleukin-6; IL-10: interleukin-10; TGF-β: transforming growth factor beta; IGF-1: insulin-like growth factor 1.

According to recent studies, oligomeric forms of α-Syn are especially neurotoxic, functioning as seeds that promote more aggregation and spread throughout the brain, thereby intensifying the degenerative process [107]. Genetic mutations (e.g., A30P, E46K, and A53T) that increase α-Syn’s ability to aggregate are among the variables that affect the aggregation process. These mutations alter the α-Syn aggregation kinetics and can cause early-onset Parkinson’s disease. Furthermore, environmental variables including exposure to toxins may cause α-Syn to undergo conformational changes that encourage aggregation [108]. Furthermore, an increasing amount of data indicates that tau and other proteins could interact with α-Syn to speed up its aggregation. The intricate pathology seen in synucleinopathies such as Parkinson’s disease and dementia with Lewy bodies may be a result of this interaction. Individual differences in clinical symptoms and diseases development have been associated with the presence of different strains of aggregated α-Syn [109] (Figure 2).

### 7.1. Microglial Activation in PD

Mechanistic findings from PD models are discussed alongside human imaging and postmortem evidence demonstrating early and sustained microglial activation. In PD, microglial dysfunction is increasingly recognized as a core pathogenic driver rather than a secondary response to dopaminergic neuron loss. Microglial activation emerges early in disease-relevant models and human tissue, precedes substantial neuronal degeneration, and directly contributes to α-synuclein propagation, neuroinflammation, and selective vulnerability of dopaminergic neurons [53]. Genetic and environmental perturbations that selectively alter microglial inflammatory and phagocytic pathways are sufficient to accelerate disease progression, underscoring the essential role of microglia in Parkinson’s disease pathophysiology [110]. Experimental models of PD demonstrate that neuroinflammatory processes are strongly influenced by microglial interactions with pathological α-synuclein (αSyn) species. Preformed fibrils (PFFs) and other pathological αSyn aggregates cause microglial activation, which raises the production of cytokines and inflammatory markers and can worsen neuronal injury [111,112]. One important modulator of this neuroinflammatory response has been shown to be the NOD2/RIPK2 signaling pathway, where αSyn binding to NOD2 promotes neurotoxic microglial activation and subsequent dopamine neuron degeneration. The inflammatory landscape in PD is further complicated by mutations such as A53T in αSyn, which increase microglial pro-inflammatory responses [113]. Microglial gene expression varies across the course of the disease, according to transcriptomic analysis, suggesting a dysregulated immune response that is linked to motor impairments and the advancement of the condition. Collectively, these results point to possible treatment targets by demonstrating the dual function of microglia in removing αSyn aggregates and causing neuroinflammation.

A key element in the pathophysiology of PD, which is typified by neuroinflammation and dopaminergic neuron loss, is the activation of microglia in the substantia nigra (SN). The NLRP3 inflammasome frequently mediates microglial activation, which worsens neurodegeneration through pro-inflammatory signaling pathways if left uncontrolled [113]. Research using MPTP models has shown that some transcription factors, such as Nfe2l2 and Runx1, drive the activation of microglia, which results in a pro-inflammatory phenotype. Targeting interferon regulatory factor 8 (IRF8) may also reduce microglial activation and enhance neuronal survival, since its overexpression in microglia has been connected to elevated neuroinflammation [114]. Additionally, microRNAs have a major impact on microglial activity modulation, affecting both the inflammatory response and microglial activation in PD [115]. A similar bidirectional relationship between neuroinflammation and oxidative stress is evident in PD. Activated microglia in the substantia nigra produce excessive reactive oxygen species, which enhance α-synuclein aggregation and neuronal vulnerability. Oxidative stress, in turn, promotes microglial inflammasome activation and the release of pro-inflammatory cytokines, further amplifying dopaminergic neuron degeneration. Genetic and toxin-based models of PD demonstrate that disrupting microglial oxidative pathways attenuates neuroinflammation and slows disease progression, underscoring oxidative stress as an active driver of microglia-mediated neurodegeneration [110,116]. The potential for customized immunomodulation in the treatment of PD has been highlighted by therapeutic approaches that have demonstrated promise in lowering neuroinflammation and safeguarding dopaminergic neurons, such as microglia-specific IL-10 administration [117]. Through mechanisms including phagocytosis and the production of pro-inflammatory cytokines, microglia activation contributes to neuroinflammation, a characteristic of PD. With its overexpression associated with enhanced phagocytic activity in the substantia nigra, the low-affinity Fcγ receptor (FcγR) on microglia has been identified as a crucial mediator of dopaminergic neuron elimination [118,119]. In PD, phagocytic dysfunction of glial cells plays a critical role in the accumulation of α-synuclein and the loss of dopaminergic neurons. Microglia internalize extracellular α-synuclein species and damaged neuronal components; however, chronic exposure to aggregated α-synuclein impairs microglial phagocytic efficiency and promotes a neurotoxic inflammatory phenotype. Astrocytes also participate in the uptake and degradation of α-synuclein, and failure of astrocytic clearance mechanisms contributes to the propagation of pathological protein species and neuronal degeneration [110,120]. The negative consequences of microglial activation are further highlighted by the hyperreactivity of LRRK2-mutant microglia in response to neuromelanin, which causes the degeneration of healthy dopaminergic neurons [119]. Additionally, by microglial activation of matrix metalloproteinases, persistent neuroinflammation can compromise the blood–brain barrier (BBB), aggravating motor impairment and neuronal death [121]. Taken together, these findings highlight the intricate relationship between dopaminergic neuron loss and microglial activation, suggesting possible treatment targets for reducing the development of PD [32]. Transcriptomic analyses in PD similarly demonstrate pronounced microglial heterogeneity and sex-dependent immune responses. Single-cell profiling of human midbrain tissue has identified distinct inflammatory and antigen-presenting microglial states enriched in PD, with female-derived microglia showing heightened interferon and cytokine signaling compared with males. These sex-linked transcriptional differences correlate with differential vulnerability of dopaminergic neurons and may contribute to sex-specific disease incidence and progression, underscoring the importance of integrating sex as a biological variable in microglia-focused PD research [122,123]. A complicated interaction between hereditary and environmental variables affects microglial activation in PD. Pesticides, heavy metals, and air pollutants are examples of environmental exposures that have been connected to elevated neuroinflammation and microglial activation, both of which exacerbate the degeneration of dopaminergic neurons [124,125]. Although less significant on their own, genetic predispositions can intensify the impacts of certain environmental stresses, causing microglia to become more inflammatory [126,127]. Interestingly, it has been found that the NOD2/RIPK2 signaling pathway is a crucial regulator of neuroinflammation, especially when abnormal α-synuclein interactions are present. This further promotes microglial activation and neurodegeneration [126]. Furthermore, aging may cause microglial phenotypes to change toward a more neurotoxic state, making their potential neuroprotective functions more difficult to understand [128]. Therefore, understanding microglial activation in PD requires an awareness of both hereditary and environmental variables. In PD, microglial activation driven by pathological α-synuclein appears to play a more direct pathogenic role, whereas later-stage cytokine dysregulation and oxidative stress responses may represent downstream consequences of sustained neuronal injury.

### 7.2. Therapeutic Approaches Targeting Microglia in PD

Microglial-driven neuroinflammation, which is characterized by the activation of microglia, the resident immune cells of the CNS, has a major impact on the pathogenesis of Parkinson’s disease. Microglial activation in PD has historically been described using pro-inflammatory (M1-like) and anti-inflammatory (M2-like) descriptors; however, these terms represent a simplified heuristic rather than discrete phenotypes, as microglial responses instead exist along a spectrum of overlapping and reversible functional states. When M1 microglia are continuously activated, pro-inflammatory cytokines including TNF-α, IL-1β, and IL-6 are generated, which leads to the development of disease and neuronal damage. However, M2 microglia may also generate anti-inflammatory chemicals that promote tissue repair and neuroprotection, highlighting the dual role of microglia in neuroinflammation.

Previous microglia-targeting studies in PD indicate that microglia can be both injurious and protective depending on the lesion paradigm, reinforcing the need for context-specific interpretation before translation. In a progressive α-synuclein overexpression mouse model, CSF1R inhibitor-mediated microglia depletion prevented motor deficits, preserved dopaminergic neurons, and reduced α-synuclein phosphorylation, suggesting that microglia can be active drivers of lesion formation in this setting [129]. By contrast, in a 6-OHDA toxin model, microglia depletion worsened motor impairment and dopaminergic neuron loss, supporting a protective microglial contribution during early phases of acute toxin-induced injury [130].

Inflammasome-focused targeting provides an example of mechanism-specific intervention that is supported by both human-cell and in vivo evidence. Primary human microglia exposed to α-synuclein fibrils mount NLRP3 inflammasome-dependent IL-1β secretion, establishing a direct human microglial mechanism linking α-synuclein to innate immune effector activation [131]. In vivo, pharmacologic inhibition of NLRP3 using a natural product-derived inhibitor improved outcomes in a PD mouse model, supporting the translational rationale for suppressing a defined microglial inflammatory effector rather than broadly suppressing microglial [132]. Collectively, these prior investigations indicate that PD microglia are best conceptualized as pathway- and stage-dependent contributors to degeneration, and that successful therapies will likely require careful matching of target, model-relevant mechanism, and disease stage rather than uniform microglial suppression.

PD patients can use a variety of strategies to reduce microglial-driven neuroinflammation. First, microglial activation targeting can be used. It is critical to prevent M1 microglia from being activated. This can be accomplished by using pharmaceuticals that target certain signaling pathways, such as the JAK/STAT or NF-kB pathways, that are implicated in microglial activation. Afterwards, microRNA modification may be applied. Making use of microRNAs (miRNAs) that control microglial activity is a potential approach. It has been discovered that certain miRNAs can reduce neuroinflammatory responses by promoting a transition from the M1 to the M2 phenotype and inhibiting excessive inflammation. Another option is Autophagy Enhancement. Improving microglia’s autophagic functions can decrease neuroinflammation and remove misfolded proteins like α-synuclein. It has been demonstrated that autophagy protects against neurodegeneration by promoting the breakdown of toxic chemicals inside microglia.

Additionally, nutritional strategies like omega-3 fatty acids have been proposed to have anti-inflammatory qualities that may positively affect microglial activation. Exercise and Lifestyle Modifications: Frequent exercise has been linked to decreased neuroinflammation and enhanced general brain health, which may have a beneficial effect on microglial function [133]. In conclusion, to reduce microglial-driven neuroinflammation in Parkinson’s disease, a multimodal strategy that targets microglial activation, uses miRNA regulation, increases autophagy, and incorporates lifestyle modifications may be useful. Developing novel treatment approaches to halt the course of the disease and enhance patient outcomes will need an understanding of these pathways.

## 8. Conclusions

Instead of being passive reactors to neuronal damage, this review emphasizes microglia as key players in the pathophysiology of Alzheimer’s and Parkinson’s diseases. While generalized cytokine elevation seems more variable and context-dependent, convergent genetic, transcriptomic, biomarker, and functional evidence suggest that microglial programs controlling lipid metabolism, phagocytosis, and synaptic remodeling represent the most robust disease-modifying mechanisms. Microglial heterogeneity and plasticity, influenced by aging, disease stage, geographical vulnerability, and biological sex, emerge as important predictors of disease progression and therapeutic response. The most promising approaches from a therapeutic perspective are those that modify microglial state and function specifically rather than suppressing neuroinflammation broadly. Microglial engagement with amyloid pathology through Fc receptor-mediated phagocytosis and TREM2-dependent signaling is particularly relevant in Alzheimer’s disease, whereas restoring microglial handling of α-synuclein and limiting chronic activation within the substantia nigra may be most effective in Parkinson’s disease. Improved human-relevant models, the incorporation of single-cell transcriptomics and biomarkers, and targeted intervention at disease-relevant phases without interfering with vital homeostatic processes are all necessary for translational success.

## Figures and Tables

**Table 1:** Recent studies carried out to analyze role of microglia in AD.

Study	Model Used	Microglial Mechanism Studied	Direction and Nature of Change	Magnitude of Change (as Reported)	References
Egln3 and Microglial Activation in AD	APP/PS1 mice; in vitro microglial models	Regulation of microglial activation via MAPK signaling	↑ Egln3 expression in microglia; ↑ MAPK pathway activation; ↑ pro-inflammatory cytokine expression. Egln3 inhibition → ↓ microglial activation and ↓ neuroinflammation	Significant increase in MAPK signaling and inflammatory markers in AD microglia; cognitive improvement observed after Egln3 inhibition. Exact fold-change not specified	[59]
APOE4, Microglia, and AD Pathogenesis	Chimeric mice with human iPSC-derived neurons (APOE3 vs. APOE4 background)	APOE4-dependent microglial inflammatory programs	↑ Pro-inflammatory microglial subtypes in APOE4 condition; ↑ Aβ and tau pathology compared with APOE3	Two distinct pro-inflammatory microglial subpopulations identified; relative increase in pathology reported, but numerical effect sizes not provided	[60]
iMGs from AD Patients	iPSC-derived microglia-like cells from AD patients	Inflammatory and phagocytic responses	↑ Basal inflammatory cytokine production; ↑ phagocytic activity in AD-derived iMGs vs. controls	Statistically significant increases in inflammatory and phagocytic markers compared with control iMGs; exact quantitative values not reported	[61]
Cycloastragenol (CAG) and Microglial Modulation	5×FAD mice; in vitro microglial cultures	PDE4B-dependent modulation of microglial function	↑ Microglial phagocytosis; ↓ amyloid plaque burden; ↑ cognitive performance	Significant reduction in amyloid plaque load and improvement in behavioral tests; magnitude reported as statistically significant but not expressed as fold-change	[62]
Cigarette Smoke (CS) and AD Progression	AD-transgenic mice	Microglial autophagy impairment via NLRP3–mTOR signaling	↓ Microglial autophagy; ↑ NLRP3 inflammasome activation; ↑ Aβ deposition	Significant increase in amyloid deposition and inflammasome activation following CS exposure; precise quantitative values not specified	[63]

AD: Alzheimer’s disease; APP/PS1: amyloid precursor protein/presenilin-1 transgenic mouse model; MAPK: mitogen-activated protein kinase; APOE3: apolipoprotein E epsilon 3 allele; APOE4: apolipoprotein E epsilon 4 allele; Aβ: amyloid-beta; iPSC: induced pluripotent stem cell; iMGs: induced microglia-like cells; CAG: cycloastragenol; PDE4B: phosphodiesterase-4B; 5×FAD: five-familial Alzheimer’s disease transgenic mouse model; CS: cigarette smoke; NLRP3: NLR family pyrin domain-containing 3 inflammasome; mTOR: mechanistic target of rapamycin. ↑ indicates increase; ↓ indicates decrease. Directionality and magnitude are reported as described in the original studies. When exact numerical effect sizes were not provided by the authors, changes are reported qualitatively as statistically significant relative to disease- or control-matched conditions.

**Table 2:** Recent studies carried out to analyze role of microglia in PD: Summary of PD Studies Involving Microglia.

Study	Direction and Nature of Change	Mechanisms Involved	Magnitude of Change (as Reported)	Potential Therapeutic Implications	Reference
IL-17A and Microglial Exosomes in PD	↑ IL-17A levels; ↑ microglial activation; ↑ neurodegeneration	IL-17A enhances microglial activation and promotes release of microglia-derived exosomes containing ciRS-7, which dysregulates miR-7 and increases SNCA expression, contributing to neuronal injury	Significant elevation of IL-17A levels and exosomal ciRS-7 in PD models compared with controls; precise fold-change not reported	Targeting IL-17A signaling or exosomal ciRS-7 may attenuate microglia-mediated neurodegeneration in PD	[102]
Treadmill Exercise and Microglial Modulation in PD	↓ pro-inflammatory microglial markers; ↑ neuroprotective microglial markers; ↓ α-synuclein accumulation; ↑ motor performance	Exercise induces a shift in microglial phenotype toward an anti-inflammatory profile, accompanied by reduced α-synuclein burden and increased tyrosine hydroxylase expression in dopaminergic neurons	Statistically significant reductions in inflammatory markers and α-synuclein levels with corresponding improvements in motor behavior; exact quantitative values not specified	Regular exercise may serve as a non-pharmacological strategy to modulate microglial activation and protect dopaminergic neurons	[103]
Antioxidant Gene Therapy with PRDX3 in PD	↓ oxidative stress; ↓ dopaminergic neuron loss; ↑ motor function	PRDX3 overexpression maintains mitochondrial redox balance in microglia and neurons, reducing ROS-mediated cellular damage in the substantia nigra	Significant reduction in oxidative stress markers and preservation of dopaminergic neurons relative to untreated PD models; numerical effect sizes not provided	PRDX3-based antioxidant gene therapy holds potential for mitigating oxidative stress-driven neurodegeneration in PD	[104]
IL-2/Anti-IL-2 Complexes and Tregs in PD	↑ regulatory T-cell populations; ↓ microglial activation; ↓ neuroinflammation; ↓ dopaminergic neuron loss	IL-2/anti-IL-2 complexes expand Treg populations, leading to reduced pro-inflammatory cytokine production, decreased glial activation, and fewer infiltrating immune cells	Statistically significant increases in Treg numbers and corresponding reductions in neuroinflammatory markers compared with MPTP controls; exact fold-change not reported	Immunomodulation via IL-2/anti-IL-2 complexes may represent a viable strategy to suppress neuroinflammation in PD	[105]
Sleep Deprivation, Gut Microbiota, and Microglial Activation in PD	↑ microglial activation; ↑ oxidative stress; ↑ PD pathology; ↑ gut dysbiosis	Sleep deprivation alters gut microbiota composition, increasing adenosine production, which activates microglia and oxidative stress pathways in the brain	Significant exacerbation of neuroinflammation, oxidative stress, and motor deficits following sleep deprivation; magnitude reported qualitatively	Improving sleep quality and targeting gut–brain axis dysregulation may slow PD progression	[106]

PD: Parkinson’s disease; IL-17A: interleukin-17A; α-synuclein (α-Syn): alpha-synuclein; ciRS-7: circular RNA sponge for microRNA-7; miR-7: microRNA-7; SNCA: synuclein alpha gene; ROS: reactive oxygen species; PRDX3: peroxiredoxin-3; Treg: regulatory T cell; IL-2: interleukin-2; MPTP: 1-methyl-4-phenyl-1,2,3,6-tetrahydropyridine. ↑ indicates increase; ↓ indicates decrease. Directionality and magnitude are reported as described in the original studies. When exact numerical effect sizes were not provided by the authors, changes are reported qualitatively as statistically significant relative to control or disease-matched conditions.

## Data Availability

No new data were created or analyzed in this study. Data sharing is not applicable to this article.

## References

[1] Amor S., Puentes F., Baker D. (2010). Immunology PVDV-, 2010 undefined. Inflammation in Neurodegenerative Diseases.

[2] Gao J., Wang L., Liu J., Xie F., Su B., Wang X. (2017). Abnormalities of Mitochondrial Dynamics in Neurodegenerative Diseases. Antioxidants.

[3] Beal M.F. (1995). Aging, energy, and oxidative stress in neurodegenerative diseases. Ann. Neurol..

[4] United Nations (2021). World Population Ageing 2019.

[5] Zaib S., Javed H., Khan I., Jaber F., Sohail A., Zaib Z., Mehboob T., Tabassam N., Ogaly H.A. (2023). Neurodegenerative Diseases: Their Onset, Epidemiology, Causes and Treatment. ChemistrySelect.

[6] Jiang Q., Liu J., Huang S., Wang X.Y., Chen X., Liu G.-H., Ye K., Song W., Masters C.L., Wang J. (2025). Antiageing strategy for neurodegenerative diseases: From mechanisms to clinical advances. Signal Transduct. Target. Ther..

[7] John Burn D. (2010). Dementia in Parkinson’s Disease. Blue Books of Neurology.

[8] Van Dyck C.H., Swanson C.J., Aisen P., Bateman R.J., Chen C., Gee M., Kanekiyo M., Li D., Reyderman L., Cohen S. (2023). Lecanemab in Early Alzheimer’s Disease. N. Engl. J. Med..

[9] Yang T., Li S., Xu H., Walsh D.M., Selkoe D.J. (2017). Large Soluble Oligomers of Amyloid β-Protein from Alzheimer Brain Are Far Less Neuroactive Than the Smaller Oligomers to Which They Dissociate. J. Neurosci..

[10] Busche M.A., Chen X., Henning H.A., Reichwald J., Staufenbiel M., Sakmann B., Konnerth A. (2012). Critical role of soluble amyloid-β for early hippocampal hyperactivity in a mouse model of Alzheimer’s disease. Proc. Natl. Acad. Sci. USA.

[11] Brown D.S., Bernstein I.H., McClintock S.M., Munro Cullum C., Dewey R.B., Husain M., Lacritz L.H. (2016). Use of the Montreal Cognitive Assessment and Alzheimer’s Disease-8 as cognitive screening measures in Parkinson’s disease. Int. J. Geriatr. Psychiatry.

[12] Smith J.A., Das A., Ray S.K., Banik N.L. (2012). Role of pro-inflammatory cytokines released from microglia in neurodegenerative diseases. Brain Res. Bull..

[13] Zhang W., Xiao D., Mao Q., Xia H. (2023). Role of neuroinflammation in neurodegeneration development. Signal Transduct. Target. Ther..

[14] Kwon S., Iba M., Kim C., Masliah E. (2020). Immunotherapies for Aging-Related Neurodegenerative Diseases—Emerging Perspectives and New Targets. Neurotherapeutics.

[15] Badole S.P., Wankhede N.L., Tiwari P.L., Umare M.D., Taksande B.G., Upaganlawar A.B. (2021). The Importance of Mitochondrial Function in Neurons: Focus on Therapeutic Targets in Neurodegeneration. Adv. Biores..

[16] John O.O., Amarachi I.S., Chinazom A.P., Adaeze E., Kale M.B., Umare M.D., Upaganlawar A.B. (2022). Phytotherapy: A promising approach for the treatment of Alzheimer’s disease. Pharmacol. Res.-Mod. Chin. Med..

[17] Isik S., Yeman Kiyak B., Akbayir R., Seyhali R., Arpaci T. (2023). Microglia Mediated Neuroinflammation in Parkinson’s Disease. Cells.

[18] Colonna M., Butovsky O. (2017). Microglia Function in the Central Nervous System During Health and Neurodegeneration. Annu. Rev. Immunol..

[19] Mosser C.-A., Baptista S., Arnoux I., Audinat E. (2017). Microglia in CNS development: Shaping the brain for the future. Prog. Neurobiol..

[20] Roth R.H., Ding J.B. (2020). From Neurons to Cognition: Technologies for Precise Recording of Neural Activity Underlying Behavior. BME Front..

[21] Fan J., Fang L., Wu J., Guo Y., Dai Q. (2020). From Brain Science to Artificial Intelligence. Engineering.

[22] Pallarés-Moratalla C., Bergers G. (2024). The ins and outs of microglial cells in brain health and disease. Front. Immunol..

[23] Chagas Lda S., Sandre P.C., Ribeiro e Ribeiro N.C.A., Marcondes H., Oliveira Silva P., Savino W., Serfaty C.A. (2020). Environmental Signals on Microglial Function during Brain Development, Neuroplasticity, and Disease. Int. J. Mol. Sci..

[24] Gu X., Zhao Z., Chen X., Zhang L., Fang H., Zhao T., Ju S., Gao W., Qian X., Wang X. (2023). Imaging microglia surveillance during sleep-wake cycles in freely behaving mice. eLife.

[25] O’Connor J.L., Nissen J.C. (2023). The Pathological Activation of Microglia Is Modulated by Sexually Dimorphic Pathways. Int. J. Mol. Sci..

[26] Cornell J., Salinas S., Huang H.-Y., Zhou M. (2022). Microglia regulation of synaptic plasticity and learning and memory. Neural. Regen. Res..

[27] Valdearcos M., McGrath E., Brown Mayfield S., Folick A., Cheang R., Li L., Li R., Bachor T.P., Lippert R.N., Xu A.W. (2024). Microglia mediate the early-life programming of adult glucose control. broRxiv.

[28] Testen A., VanRyzin J.W., Bellinger T.J., Kim R., Wang H., Gastinger M.J., Witt E.A., Franklin J.P., Vecchiarelli H.A., Picard K. (2024). Abstinence from cocaine self-administration promotes microglia pruning of astrocytes which drives cocaine-seeking behavior. broRxiv.

[29] Haupt M., Gerner S.T., Doeppner T.R. (2024). The dual role of microglia in ischemic stroke and its modulation via extracellular vesicles and stem cells. Neuroprotection.

[30] Scheiblich H., Eikens F., Wischhof L., Opitz S., Jüngling K., Cserép C., Schmidt S.V., Lambertz J., Bellande T., Pósfai B. (2024). Microglia rescue neurons from aggregate-induced neuronal dysfunction and death through tunneling nanotubes. Neuron.

[31] Bivona G., Iemmolo M., Agnello L., Lo Sasso B., Gambino C.M., Giglio R.V., Scazzone C., Ghersi G., Ciaccio M. (2023). Microglial Activation and Priming in Alzheimer’s Disease: State of the Art and Future Perspectives. Int. J. Mol. Sci..

[32] Gao C., Jiang J., Tan Y., Chen S. (2023). Microglia in neurodegenerative diseases: Mechanism and potential therapeutic targets. Signal Transduct. Target. Ther..

[33] Pesämaa I., Müller S.A., Robinson S., Darcher A., Paquet D., Zetterberg H., Lichtenthaler S.F., Haass C. (2023). A microglial activity state biomarker panel differentiates FTD-granulin and Alzheimer’s disease patients from controls. Mol. Neurodegener..

[34] Liu G., Zhang H. (2024). The Role of Lactylation in Regulating Microglial Inflammation in PTSD. Discov. Med..

[35] Fiebich B.L., Batista C.R.A., Saliba S.W., Yousif N.M., de Oliveira A.C.P. (2018). Role of Microglia TLRs in Neurodegeneration. Front. Cell. Neurosci..

[36] Ulland T.K., Song W.M., Huang S.C.-C., Ulrich J.D., Sergushichev A., Beatty W.L., Loboda A.A., Zhou Y., Cairns N.J., Kambal A. (2017). TREM2 Maintains Microglial Metabolic Fitness in Alzheimer’s Disease. Cell.

[37] Maphis N., Xu G., Kokiko-Cochran O.N., Cardona A.E., Ransohoff R.M., Lamb B.T., Bhaskar K. (2015). Loss of tau rescues inflammation-mediated neurodegeneration. Front. Neurosci..

[38] Konishi H., Okamoto T., Hara Y., Komine O., Tamada H., Maeda M., Osako F., Kobayashi M., Nishiyama A., Kataoka Y. (2020). Astrocytic phagocytosis is a compensatory mechanism for microglial dysfunction. EMBO J..

[39] Moehle M.S., West A.B. (2015). M1 and M2 immune activation in Parkinson’s Disease: Foe and ally?. Neuroscience.

[40] Lan Z., Tan F., He J., Liu J., Lu M., Hu Z., Zhuo Y., Liu J., Tang X., Jiang Z. (2024). Curcumin-primed olfactory mucosa-derived mesenchymal stem cells mitigate cerebral ischemia/reperfusion injury-induced neuronal PANoptosis by modulating microglial polarization. Phytomedicine.

[41] Zhang J., Zhang Y., Wang J., Xia Y., Zhang J., Chen L. (2024). Recent advances in Alzheimer’s disease: Mechanisms, clinical trials and new drug development strategies. Signal Transduct. Target. Ther..

[42] Shankar G.M., Li S., Mehta T.H., Garcia-Munoz A., Shepardson N.E., Smith I., Brett F.M., Farrell M.A., Rowan M.J., Lemere C.A. (2008). Amyloid-β protein dimers isolated directly from Alzheimer’s brains impair synaptic plasticity and memory. Nat. Med..

[43] Gu L., Guo Z. (2013). Alzheimer’s Aβ42 and Aβ40 peptides form interlaced amyloid fibrils. J. Neurochem..

[44] Wang Q., Xie C. (2022). Microglia activation linking amyloid-β drive tau spatial propagation in Alzheimer’s disease. Front. Neurosci..

[45] Tosun D., Veitch D., Aisen P., Jack C.R., Jagust W.J., Petersen R.C., Saykin A.J., Bollinger J., Ovod V., Mawuenyega K.G. (2021). Detection of β-amyloid positivity in Alzheimer’s Disease Neuroimaging Initiative participants with demographics, cognition, MRI and plasma biomarkers. Brain Commun..

[46] DeTure M.A., Dickson D.W. (2019). The neuropathological diagnosis of Alzheimer’s disease. Mol. Neurodegener..

[47] Busche M.A., Hyman B.T. (2020). Synergy between amyloid-β and tau in Alzheimer’s disease. Nat. Neurosci..

[48] Sheng J.G., Ito K., Skinner R.D., Mrak R.E., Rovnaghi C.R., van Eldik L.J., Griffin W.T. (1996). In vivo and in vitro evidence supporting a role for the inflammatory cytokine interleukin-1 as a driving force in Alzheimer pathogenesis. Neurobiol. Aging.

[49] Griffin W.S.T., Liu L., Li Y., Mrak R.E., Barger S.W. (2006). Interleukin-1 mediates Alzheimer and Lewy body pathologies. J. Neuroinflamm..

[50] Sims R., van der Lee S.J., Naj A.C., Bellenguez C., Badarinarayan N., Jakobsdottir J., Kunkle B.W., Boland A. (2017). Rare coding variants in PLCG2, ABI3, and TREM2 implicate microglial-mediated innate immunity in Alzheimer’s disease. Nat. Genet..

[51] Guerreiro R., Wojtas A., Bras J., Carrasquillo M., Rogaeva E., Majounie E., Cruchaga C., Sassi C., Kauwe J.S.K., Younkin S. (2013). *TREM2* Variants in Alzheimer’s Disease. N Engl. J. Med..

[52] Ewers M., Franzmeier N., Suárez-Calvet M., Morenas-Rodriguez E., Caballero M.A.A., Kleinberger G., Piccio L., Deming Y., Dichgans M., Trojanowski J.Q. (2019). Increased soluble TREM2 in cerebrospinal fluid is associated with reduced cognitive and clinical decline in Alzheimer’s disease. Sci. Transl. Med..

[53] Gerhard A., Pavese N., Hotton G., Turkheimer F., Es M., Hammers A., Eggert K., Oertel W., Banati R.B., Brooks D.J. (2006). In vivo imaging of microglial activation with [11C](R)-PK11195 PET in idiopathic Parkinson’s disease. Neurobiol. Dis..

[54] Keren-Shaul H., Spinrad A., Weiner A., Matcovitch-Natan O., Dvir-Szternfeld R., Ulland T.K., David E., Baruch K., Lara-Astaiso D., Toth B. (2017). A Unique Microglia Type Associated with Restricting Development of Alzheimer’s Disease. Cell.

[55] Vandenbark A.A., Offner H., Matejuk S., Matejuk A. (2021). Microglia and astrocyte involvement in neurodegeneration and brain cancer. J Neuroinflamm..

[56] Valiukas Z., Tangalakis K., Apostolopoulos V., Feehan J. (2025). Microglial activation states and their implications for Alzheimer’s Disease. J. Prev. Alzheimers Dis..

[57] Liddelow S.A., Guttenplan K.A., Clarke L.E., Bennett F.C., Bohlen C.J., Schirmer L., Bennett M.L., Münch A.E., Chung W.S., Peterson T.C. (2017). Neurotoxic reactive astrocytes are induced by activated microglia. Nature.

[58] Zhao X., Zhang S., Sanders A.R., Duan J. (2023). Brain Lipids and Lipid Droplet Dysregulation in Alzheimer’s Disease and Neuropsychiatric Disorders. Complex Psychiatry.

[59] Guan J., Wu P., Liu M., Jiang C., Meng X., Wu X., Lu M., Fan Y., Gan L. (2025). Egln3 expression in microglia enhances the neuroinflammatory responses in Alzheimer’s disease. Brain Behav. Immun..

[60] Rao A., Chen N., Kim M.J., Blumenfeld J., Yip O., Liang Z., Shostak D., Hao Y., Nelson M.R., Koutsodendris N. (2025). Microglia depletion reduces human neuronal APOE4-related pathologies in a chimeric Alzheimer’s disease model. Cell Stem Cell.

[61] Gonul C.P., Kiser C., Yaka E.C., Oz D., Hunerli D., Yerlikaya D., Olcum M., Keskinoglu P., Yener G., Genc S. (2025). Microglia-like cells from patient monocytes demonstrate increased phagocytic activity in probable Alzheimer’s disease. Mol. Cell. Neurosci..

[62] Weng W., Lin B., Zheng J., Sun Y., Li Z., Chen X., Wang Y., Pan X. (2025). Novel application of cycloastragenol target microglia for the treatment of Alzheimer’s disease: Evidence from single-cell analysis, network pharmacology and experimental assessment. Phytomedicine.

[63] Wang H., Xia H., Bai J., Wang Z., Wang Y., Lin J., Cheng C., Chen W., Zhang J., Zhang Q. (2025). H4K12 lactylation-regulated NLRP3 is involved in cigarette smoke-accelerated Alzheimer-like pathology through mTOR-regulated autophagy and activation of microglia. J. Hazard. Mater..

[64] Vassileff N., Spiers J.G., Bamford S.E., Lowe R.G.T., Datta K.K., Pigram P.J., Hill A.F. (2024). Microglial activation induces nitric oxide signalling and alters protein S-nitrosylation patterns in extracellular vesicles. J. Extracell. Vesicles.

[65] Malek N., Gladysz R., Stelmach N., Drag M. (2024). Targeting Microglial Immunoproteasome: A Novel Approach in Neuroinflammatory-Related Disorders. ACS Chem. Neurosci..

[66] Valerio R.R., Santos Á.R., Nóbrega A.H.L., Martins R., De Felice F.G., Ferreira S.T., Savino W., Bonomo A., Bernardi A., Frozza R.L. (2026). Innate Immune Tolerance Regulates Microglia Response to Aβ Oligomers. J. Neurochem..

[67] Wang W., Zhao F., Ma X., Perry G., Zhu X. (2020). Mitochondria dysfunction in the pathogenesis of Alzheimer’s disease: Recent advances. Mol. Neurodegener..

[68] Block M.L., Zecca L., Hong J.-S. (2007). Microglia-mediated neurotoxicity: Uncovering the molecular mechanisms. Nat. Rev. Neurosci..

[69] Heneka M.T., Kummer M.P., Stutz A., Delekate A., Schwartz S., Vieira-Saecker A., Griep A., Axt D., Remus A., Tzeng T.-C. (2013). NLRP3 is activated in Alzheimer’s disease and contributes to pathology in APP/PS1 mice. Nature.

[70] Forloni G. (2023). Alpha Synuclein: Neurodegeneration and Inflammation. Int. J. Mol. Sci..

[71] Luo H., Xiang Y., Qu X., Liu H., Liu C., Li G., Han L., Qin X. (2019). Apelin-13 Suppresses Neuroinflammation Against Cognitive Deficit in a Streptozotocin-Induced Rat Model of Alzheimer’s Disease Through Activation of BDNF-TrkB Signaling Pathway. Front. Pharmacol..

[72] Vainchtein I.D., Chin G., Cho F.S., Kelley K.W., Miller J.G., Chien E.C., Liddelow S.A., Nguyen P.T., Nakao-Inoue H., Dorman L.C. (2018). Astrocyte-derived interleukin-33 promotes microglial synapse engulfment and neural circuit development. Science.

[73] Clarke L.E., Liddelow S.A., Chakraborty C., Münch A.E., Heiman M., Barres B.A. (2018). Normal aging induces A1-like astrocyte reactivity. Proc. Natl. Acad. Sci. USA.

[74] Ewers M., Biechele G., Suárez-Calvet M., Sacher C., Blume T., Morenas-Rodriguez E., Deming Y., Piccio L., Cruchaga C., Kleinberger G. (2020). Higher CSF sTREM2 and microglia activation are associated with slower rates of beta-amyloid accumulation. EMBO Mol. Med..

[75] Fang S., Wu Z., Guo Y., Zhu W., Wan C., Yuan N., Chen J., Hao W., Mo X., Guo X. (2023). Roles of microglia in adult hippocampal neurogenesis in depression and their therapeutics. Front. Immunol..

[76] Taddei R.N., Perbet R., Mate de Gerando A., Wiedmer A.E., Sanchez-Mico M., Connors Stewart T., Gaona A., Melloni A., Amaral A.C., Duff K. (2023). Tau Oligomer–Containing Synapse Elimination by Microglia and Astrocytes in Alzheimer Disease. JAMA Neurol..

[77] Vicario R., Fragkogianni S., Weber L., Lazarov T., Hu Y., Hayashi S.Y., Craddock B., Socci N.D., Alberdi A., Baako A. (2024). A microglia clonal inflammatory disorder in Alzheimer’s Disease. eLife.

[78] Del-Aguila J.L., Li Z., Dube U., Mihindukulasuriya K.A., Budde J.P., Fernandez M.V., Ibanez L., Bradley J., Wang F., Bergmann K. (2019). A single-nuclei RNA sequencing study of Mendelian and sporadic AD in the human brain. Alzheimers Res. Ther..

[79] Mathys H., Davila-Velderrain J., Peng Z., Gao F., Mohammadi S., Young J.Z., Menon M., He L., Abdurrob F., Jiang X. (2019). Single-cell transcriptomic analysis of Alzheimer’s disease. Nature.

[80] Johnson N.R., Yuan P., Castillo E., Lopez T.P., Yue W., Bond A., Rivera B.M., Sullivan M.C., Hirouchi M., Giles K. (2023). CSF1R inhibitors induce a sex-specific resilient microglial phenotype and functional rescue in a tauopathy mouse model. Nat. Commun..

[81] Ganz T., Fainstein N., Elad A., Lachish M., Goldfarb S., Einstein O., Ben-Hur T. (2022). Microbial pathogens induce neurodegeneration in Alzheimer’s disease mice: Protection by microglial regulation. J. Neuroinflamm..

[82] Sahara N., Yanai R. (2023). Limitations of human tau-expressing mouse models and novel approaches of mouse modeling for tauopathy. Front Neurosci.

[83] Gamache J., Benzow K., Forster C., Kemper L., Hlynialuk C., Furrow E., Ashe K.H., Koob M.D. (2019). Factors other than hTau overexpression that contribute to tauopathy-like phenotype in rTg4510 mice. Nat. Commun..

[84] Huat T.J., Camats-Perna J., Newcombe E.A., Valmas N., Kitazawa M., Medeiros R. (2019). Metal Toxicity Links to Alzheimer’s Disease and Neuroinflammation. J. Mol. Biol..

[85] von Bernhardi R., Eugenín-von Bernhardi L., Eugenín J. (2015). Microglial cell dysregulation in brain aging and neurodegeneration. Front. Aging Neurosci..

[86] Lin M., Yu J.-X., Zhang W.-X., Lao F.-X., Huang H.-C. (2024). Roles of TREM2 in the Pathological Mechanism and the Therapeutic Strategies of Alzheimer’s Disease. J. Prev. Alzheimers Dis..

[87] Li L., Zheng X., Ma H., Zhu M., Li X., Sun X., Feng X. (2025). TREM2 in Neurodegenerative Diseases: Mechanisms and Therapeutic Potential. Cells.

[88] Schlepckow K., Monroe K.M., Kleinberger G., Cantuti-Castelvetri L., Parhizkar S., Xia D., Willem M., Werner G., Pettkus N., Brunner B. (2020). Enhancing protective microglial activities with a dual function TREM_2_ antibody to the stalk region. EMBO Mol. Med..

[89] Spangenberg E., Severson P.L., Hohsfield L.A., Crapser J., Zhang J., Burton E.A., Zhang Y., Spevak W., Lin J., Phan N.Y. (2019). Sustained microglial depletion with CSF1R inhibitor impairs parenchymal plaque development in an Alzheimer’s disease model. Nat. Commun..

[90] Zhao N., Qiao W., Li F., Ren Y., Zheng J., Martens Y.A., Wang X., Li L., Liu C.-C., Chen K. (2022). Elevating microglia TREM2 reduces amyloid seeding and suppresses disease-associated microglia. J. Exp. Med..

[91] Jain N., Lewis C.A., Ulrich J.D., Holtzman D.M. (2023). Chronic TREM2 activation exacerbates Aβ-associated tau seeding and spreading. J. Exp. Med..

[92] Long H., Simmons A., Mayorga A., Burgess B., Nguyen T., Budda B., Rychkova A., Rhinn H., Tassi I., Ward M. (2024). Preclinical and first-in-human evaluation of AL002, a novel TREM2 agonistic antibody for Alzheimer’s disease. Alzheimers Res. Ther..

[93] Guo S., Wang H., Yin Y. (2022). Microglia Polarization from M1 to M2 in Neurodegenerative Diseases. Front. Aging Neurosci..

[94] Maezawa I., Nguyen H.M., Di Lucente J., Jenkins D.P., Singh V., Hilt S., Kim K., Rangaraju S., I Levey A., Wulff H. (2018). Kv1.3 inhibition as a potential microglia-targeted therapy for Alzheimer’s disease: Preclinical proof of concept. Brain.

[95] Sevigny J., Chiao P., Bussière T., Weinreb P.H., Williams L., Maier M., Dunstan R., Salloway S., Chen T., Ling Y. (2016). The antibody aducanumab reduces Aβ plaques in Alzheimer’s disease. Nature.

[96] Mintun M.A., Lo A.C., Duggan Evans C., Wessels A.M., Ardayfio P.A., Andersen S.W., Shcherbinin S., Sparks J., Sims J.R., Brys M. (2021). Donanemab in Early Alzheimer’s Disease. N. Engl. J. Med..

[97] Ulrich J.D., Ulland T.K., Colonna M., Holtzman D.M. (2017). Elucidating the Role of TREM2 in Alzheimer’s Disease. Neuron.

[98] Wang Y., Cella M., Mallinson K., Ulrich J.D., Young K.L., Robinette M.L., Gilfillan S., Krishnan G.M., Sudhakar S., Zinselmeyer B.H. (2015). TREM2 Lipid Sensing Sustains the Microglial Response in an Alzheimer’s Disease Model. Cell.

[99] Sims J.R., Zimmer J.A., Evans C.D., Lu M., Ardayfio P., Sparks J., Wessels A.M., Shcherbinin S., Wang H., Nery E.S.M. (2023). Donanemab in Early Symptomatic Alzheimer Disease. JAMA.

[100] Gómez-Benito M., Granado N., García-Sanz P., Michel A., Dumoulin M., Moratalla R. (2020). Modeling Parkinson’s Disease with the Alpha-Synuclein Protein. Front. Pharmacol..

[101] Yi S., Wang L., Wang H., Ho M.S., Zhang S. (2022). Pathogenesis of α-Synuclein in Parkinson’s Disease: From a Neuron-Glia Crosstalk Perspective. Int. J. Mol. Sci..

[102] Xu F.-F., Liu Z., Fang X.-X., Cao B.-B., Huang Y., Peng Y.-P., Qiu Y.-H. (2025). Microglia-derived exosomal ciRS-7 mediates IL-17A effect of promoting neurodegeneration via miR-7 and SNCA targets in an experimental Parkinson’s disease. Int. Immunopharmacol..

[103] Kumar D., Kumar R., Janrao S., Sharma V., Begum N., Fernandes V., Khatri D.K. (2025). Treadmill exercise mitigates rotenone-induced neuroinflammation and α-synuclein level in a mouse model of Parkinson’s disease. Brain Res..

[104] Villa-Cedillo S.A., Acosta-Espinoza E.J., Soto-Domínguez A., Rodríguez-Rocha H., Montes-de-Oca-Saucedo C.R., García-García A., Loera-Arias M.d.J., Ríos-Vazquez C.S., Sánchez-Torres G., Valdés J. (2025). Antioxidant PRDX3 gene therapy protects brain cells and prevents neurodegeneration in an animal model of Parkinson’s disease. Neuropeptides.

[105] Li L., Gao W., Ren N., Chen L. (2025). IL-2/anti-IL-2 complexes attenuates neuroinflammation and neurodegeneration in mice of experimental Parkinson’s disease. Brain Res. Bull..

[106] Zhu W., Hu Y., Shi Y., Bao H., Cheng X., Jiang M., Peng Z., Song J., Fang F., Jian C. (2025). Sleep deprivation accelerates Parkinson’s disease via modulating gut microbiota associated microglial activation and oxidative stress. Microbiol. Res..

[107] Du X., Xie X., Liu R. (2020). The Role of α-Synuclein Oligomers in Parkinson’s Disease. Int. J. Mol. Sci..

[108] Krasnoslobodtsev A.V., Volkov I.L., Asiago J.M., Hindupur J., Rochet J.-C., Lyubchenko Y.L. (2013). α-Synuclein Misfolding Assessed with Single Molecule AFM Force Spectroscopy: Effect of Pathogenic Mutations. Biochemistry.

[109] Clinton L.K., Blurton-Jones M., Myczek K., Trojanowski J.Q., LaFerla F.M. (2010). Synergistic Interactions between Aβ, Tau, and α-Synuclein: Acceleration of Neuropathology and Cognitive Decline. J. Neurosci..

[110] Kim J., Byun J.-W., Choi I., Kim B., Jeong H.-K., Jou I., Joe E. (2013). PINK1 Deficiency Enhances Inflammatory Cytokine Release from Acutely Prepared Brain Slices. Exp. Neurobiol..

[111] Krzisch M., Yuan B., Chen W., Osaki T., Fu D., Garrett-Engele C.M., Svoboda D.S., Andrykovich K.R., Gallagher M.D., Sur M. (2024). The A53T Mutation in α-Synuclein Enhances Proinflammatory Activation in Human Microglia Upon Inflammatory Stimulus. Biol. Psychiatry.

[112] Stoll A.C., Kemp C.J., Patterson J.R., Howe J.W., Steece-Collier K., Luk K.C., Sortwell C.E., Benskey M.J. (2024). Neuroinflammatory gene expression profiles of reactive glia in the substantia nigra suggest a multidimensional immune response to alpha synuclein inclusions. Neurobiol. Dis..

[113] Pike A.F., Longhena F., Faustini G., van Eik J.-M., Gombert I., Herrebout M.A.C., Fayed M.M.H.E., Sandre M., Varanita T., Teunissen C.E. (2022). Dopamine signaling modulates microglial NLRP3 inflammasome activation: Implications for Parkinson’s disease. J. Neuroinflamm..

[114] Ma W., Huang G., Wang Z., Wang L., Gao Q. (2023). IRF7: Role and regulation in immunity and autoimmunity. Front. Immunol..

[115] Zheng D., Chen J. (2025). MicroRNAs in Parkinson’s disease: From pathogenesis to diagnostics and therapeutic strategies. Neuroscience.

[116] Gao H.-M., Zhou H., Hong J.-S. (2012). NADPH oxidases: Novel therapeutic targets for neurodegenerative diseases. Trends Pharmacol. Sci..

[117] Bido S., Nannoni M., Muggeo S., Gambarè D., Ruffini G., Bellini E., Passeri L., Iaia S., Luoni M., Provinciali M. (2024). Microglia-specific *IL-10* gene delivery inhibits neuroinflammation and neurodegeneration in a mouse model of Parkinson’s disease. Sci. Transl. Med..

[118] Brandli A., Vessey K.A., Fletcher E.L. (2024). The contribution of pattern recognition receptor signalling in the development of age related macular degeneration: The role of toll-like-receptors and the NLRP3-inflammasome. J. Neuroinflamm..

[119] Oun A., Sabogal-Guaqueta A.M., Galuh S., Alexander A., Kortholt A., Dolga A.M. (2022). The multifaceted role of LRRK2 in Parkinson’s disease: From human iPSC to organoids. Neurobiol. Dis..

[120] Lee H.-J., Suk J.-E., Patrick C., Bae E.-J., Cho J.-H., Rho S., Hwang D., Masliah E., Lee S.-J. (2010). Direct Transfer of α-Synuclein from Neuron to Astroglia Causes Inflammatory Responses in Synucleinopathies. J. Biol. Chem..

[121] Ruan Z., Zhang D., Huang R., Sun W., Hou L., Zhao J., Wang Q. (2022). Microglial Activation Damages Dopaminergic Neurons through MMP-2/-9-Mediated Increase of Blood-Brain Barrier Permeability in a Parkinson’s Disease Mouse Model. Int. J. Mol. Sci..

[122] Smajić S., Prada-Medina C.A., Landoulsi Z., Ghelfi J., Delcambre S., Dietrich C., Jarazo J., Henck J., Balachandran S., Pachchek S. (2022). Single-cell sequencing of human midbrain reveals glial activation and a Parkinson-specific neuronal state. Brain.

[123] Lopez-Lee C., Kodama L., Gan L. (2022). Sex Differences in Neurodegeneration: The Role of the Immune System in Humans. Biol. Psychiatry.

[124] Sarkar S. (2021). Mechanism of Gene-Environment Interactions Driving Glial Activation in Parkinson’s Diseases. Curr. Environ. Health Rep..

[125] Polito M.D., Souza D.B., Casonatto J., Farinatti P. (2016). Acute effect of caffeine consumption on isotonic muscular strength and endurance: A systematic review and meta-analysis. Sci. Sports.

[126] Seo B.A., Kwon S.-H., Kim D., Kim H.-B., Ma S.-X., Gadhave K., Burgess N., Mao X., Rosenthal L.S., Redding-Ochoa J. (2024). Pathologic α-Synuclein-NOD2 Interaction and RIPK2 Activation Drives Microglia-Induced Neuroinflammation in Parkinson’s Disease. broRxiv.

[127] Polito R., La Torre M.E., Moscatelli F., Cibelli G., Valenzano A., Panaro M.A., Monda M., Messina A., Monda V., Pisanelli D. (2023). The Ketogenic Diet and Neuroinflammation: The Action of Beta-Hydroxybutyrate in a Microglial Cell Line. Int. J. Mol. Sci..

[128] L’Episcopo F., Tirolo C., Serapide M.F., Caniglia S., Testa N., Leggio L., Vivarelli S., Iraci N., Pluchino S., Marchetti B. (2018). Microglia Polarization, Gene-Environment Interactions and Wnt/β-Catenin Signaling: Emerging Roles of Glia-Neuron and Glia-Stem/Neuroprogenitor Crosstalk for Dopaminergic Neurorestoration in Aged Parkinsonian Brain. Front. Aging Neurosci..

[129] Zhang Z., Niu K., Huang T., Guo J., Xarbat G., Gong X., Gao Y., Liu F., Cheng S., Su W. (2025). Microglia depletion reduces neurodegeneration and remodels extracellular matrix in a mouse Parkinson’s disease model triggered by α-synuclein overexpression. npj Park. Dis..

[130] Pereira C.P.M., Francis-Oliveira J., Singulani M.P., Ferreira A.F.F., Britto L.R.G. (2023). Microglial depletion exacerbates motor impairment and dopaminergic neuron loss in a 6-OHDA model of Parkinson’s disease. J. Neuroimmunol..

[131] Pike A.F., Varanita T., Herrebout M.A.C., Plug B.C., Kole J., Musters R.J.P., Teunissen C.E., Hoozemans J.J.M., Bubacco L., Veerhuis R. (2021). α-Synuclein evokes NLRP3 inflammasome-mediated IL-1β secretion from primary human microglia. Glia.

[132] Li W., Zhang J., Chen Q., Luo B., Zhou B., Wang J., Yang Y., Liu Y., Wen S., Kong D.-X. (2025). Treatment of Parkinson’s Disease with an Anti-Inflammasome NLRP3 Inhibitor Derived from a Natural Product. ACS Pharmacol. Transl. Sci..

[133] Chang B., Jiang Y., Feng C., Li B., Mei J., Niu C. (2025). Effect of purine diet on prognosis of deep brain stimulation for Parkinson’s disease. Food Sci. Hum. Wellness.

